# Risk stratification for early bacteremia after living donor liver transplantation: a retrospective observational cohort study

**DOI:** 10.1186/s12893-019-0658-6

**Published:** 2020-03-12

**Authors:** Jaesik Park, Bae Wook Kim, Ho Joong Choi, Sang Hyun Hong, Chul Soo Park, Jong Ho Choi, Min Suk Chae

**Affiliations:** 1 Department of Anesthesiology and Pain medicine, Seoul St. Mary’s Hospital, College of Medicine, The Catholic University of Korea, 222, Banpo-daero, Seocho-gu, Seoul, 06591 Republic of Korea; 2 Department of Surgery, Seoul St. Mary’s Hospital, College of Medicine, The Catholic University of Korea, Seoul, Republic of Korea

**Keywords:** Bacteremia, Inflammation, Psoas muscles, Primary graft dysfunction, Renal replacement therapy

## Abstract

**Background:**

This study investigated perioperative clinical risk factors for early post-transplant bacteremia in patients undergoing living donor liver transplantation (LDLT). Additionally, postoperative outcomes were compared between patients with and without early post-transplant bacteremia.

**Methods:**

Clinical data of 610 adult patients who underwent elective LDLT between January 2009 and December 2018 at Seoul St. Mary’s Hospital were retrospectively collected. The exclusion criteria included overt signs of infection within 1 month before surgery. A total of 596 adult patients were enrolled in this study. Based on the occurrence of a systemic bacterial infection after surgery, patients were classified into non-infected and infected groups.

**Results:**

The incidence of bacteremia at 1 month after LDLT was 9.7% (57 patients) and *Enterococcus faecium* (31.6%) was the most commonly cultured bacterium in the blood samples. Univariate analysis showed that preoperative psoas muscle index (PMI), model for end-stage disease score, utility of continuous renal replacement therapy (CRRT), ascites, C-reactive protein to albumin ratio, neutrophil to lymphocyte ratio (NLR), platelet to lymphocyte ratio, and sodium level, as well as intraoperative post-reperfusion syndrome, mean central venous pressure, requirement for packed red blood cells and fresh frozen plasma, hourly fluid infusion and urine output, and short-term postoperative early allograft dysfunction (EAD) were associated with the risk of early post-transplant bacteremia. Multivariate analysis revealed that PMI, the CRRT requirement, the NLR, and EAD were independently associated with the risk of early post-transplant bacteremia (area under the curve: 0.707; 95% confidence interval: 0.667–0.745; *p* < 0.001). The overall survival rate was better in the non-infected patient group. Among patients with bacteremia, anti-bacterial treatment was unable to resolve infection in 34 patients, resulting in an increased risk of patient mortality. Among the factors included in the model, EAD was significantly correlated with non-resolving infection.

**Conclusions:**

We propose a prognostic model to identify patients at high risk for a bloodstream bacterial infection; furthermore, our findings support the notion that skeletal muscle depletion, CRRT requirement, systemic inflammatory response, and delayed liver graft function are associated with a pathogenic vulnerability in cirrhotic patients who undergo LDLT.

## Introduction

Living donor liver transplantation (LDLT) has been widely accepted as an appropriate alternative treatment in patients with end-stage liver disease (ESLD), which is necessary due to the imbalance between graft demand and supply [1]. Because of the importance of partial liver grafts in LDLT, they must meet the metabolic demands and grow to a size appropriate for the patient’s body [2]. Patient and graft survival has improved progressively with advances in surgical techniques and perioperative critical care. However, infection remains a major cause of morbidity and mortality, and can further aggravate cirrhotic complications, such as refractory ascites and/or hepatorenal syndrome, in patients with ESLD who undergo liver transplantation (LT) [3, 4].

The overall incidence of infection, including bacteremia, urinary tract infection, and pneumonia, is higher in patients with versus without cirrhosis [5]. Additionally, the infection risk is about 10-fold higher in cirrhotic patients than in the general population [6]. Bacterial infections are predominant in LT patients (accounting for up to 70% of all infections), followed by fungal and viral infections. The infection risk varies with postoperative time course [3]. During the early post-transplant period (i.e., ≤ 1 month), bacteria pathogens are frequently isolated from blood samples, and patients with a bloodstream infection have a higher early mortality rate than those without such an infection [7]. Potential causes of infectious susceptibility in patients with ESLD include impaired immune function in the local liver, systemic immunity, and breakdown of the mucocutaneous barrier (which causes bacteria and the products thereof to translocate from the intestines to the central circulation) [8, 9]. Therefore, because of the high risk of sepsis in patients with ESLD, early risk stratification of vulnerable patients undergoing LDLT is of paramount importance.

This study investigated perioperative clinical risk factors for early post-transplant bacteremia in patients undergoing LDLT. Additionally, postoperative outcomes, including overall patient survival, were compared between patients with and without early post-transplant bacteremia.

## Patients and methods

### Ethical considerations

The present study on LDLT patients was approved by the Institutional Review Board of Seoul St. Mary’s Hospital Ethics Committee (KC19RESI0214; April 15, 2019), and was performed according to the principles of the Declaration of Helsinki. The requirement for informed consent was waived due to the retrospective nature of the study.

### Study population

Clinical data of 610 adult patients (aged ≥19 years) who underwent elective LDLT between January 2009 and December 2018 at Seoul St. Mary’s Hospital were retrospectively collected from the electronic medical records system. The exclusion criteria included overt signs of infection within 1 month before surgery, with the infection source identified by blood, urine, ascites, or sputum culture; chest X-ray and/or computed tomography (CT) images of the lung or abdomen; and the clinical presentation [10], to decrease the preoperative impact of infection sources on newly occurring post-transplant bacteremia during the early period. Ultimately, 596 adult patients were enrolled in this study.

### Living donor liver transplantation

The surgical procedure and anesthetic protocol used herein have been described in detail previously [11, 12]. Briefly, the piggyback surgical technique was performed using the right liver lobe with reconstruction of the middle hepatic vein. After completion of hepatic vascular and ductal anastomoses, the patency of hepatic vascular flow was confirmed using Doppler ultrasonography. Balanced anesthesia was applied for several hemodynamic monitoring modalities, including radial arterial and central venous cannulation, which were performed using a sterile technique. Blood products were transfused according to laboratory measurements or thromboelastography. Immediately after surgery, hemodynamically stable and mentally alert patients were extubated in the operating room to prevent unnecessary mechanical ventilation [13].

Intravenous cefobactam (1 g; cefoperazone and sulbactam; Hanmi Pharm, Seoul, Republic of Korea) was infused immediately before the skin incision and graft reperfusion in the operating room, and was subsequently administered every 12 h during postoperative day (POD) 6; intravenous Penbrex (2 g; ampicillin; Yungjin Pharm, Seoul, Republic of Korea) was infused postoperatively every 6 h between the day of surgery and POD 4.

In patients with hepatitis B, 10,000 IU of intravenous hepabulin SN (hepatitis B immunoglobulin; SK Plasma, Seoul, Republic of Korea) was infused immediately before graft reperfusion in the operating room, and subsequently administered once during POD 7. The hepabulin SN was gradually tapered during the first week after surgery. Viread Tab (300 mg tenofovir disoproxil fumarate; Gilead Science, Inc., Foster City, CA, USA) was applied once daily after surgery.

A triple immunosuppression drug regimen, including tacrolimus (Astellas, Tokyo, Japan), mycophenolate mofetil (Chong Kun Dang Pharm, Seoul, Republic of Korea), and methylprednisolone (Reyon Pharm, Seoul, Republic of Korea) was administered after surgery. The initial dose of tacrolimus was 1 mg. Subsequently, the infusion dose was modified based on trough levels (between 7 and 10 ng·mL^− 1^) for the first month after surgery, and gradually tapered to between 5 and 7 ng·mL^− 1^ thereafter. Methylprednisolone (250 mg) was administered immediately before graft reperfusion and then tapered gradually. Mycophenolate mofetil (500 mg) was initiated and then withdrawn at 3–6 months after surgery. Basiliximab (interleukin-2 receptor antagonist; Novartis, Basel, Switzerland) was administered on the day of LDLT prior to the surgery, and on POD 4.

According to our hospital desensitization protocol for ABO-incompatible grafts, patients were intravenously infused with rituximab (375 mg.m^− 2^) (Mabthera; Roche, Basel, Switzerland) at 2 weeks before surgery, and plasmapheresis using fresh frozen plasma (FFP) was instituted in blood type AB^+^ patients. The plasmapheresis was consistently applied to reach an acceptable isohemagglutinin titer (≤ 1:32) prior to the surgery.

### Early post-transplant bacteremia

Blood cultures were obtained regularly (once every 3 days) during the first month after surgery. Two pairs of aerobic and anaerobic bottles (BACTEC Plus Aerobic and Anaerobic Lytic media; Becton, Dickinson and Co., Franklin Lakes, NJ, USA) were used, and incubated for at least 5 days. Isolated bacteria were analyzed by standard microbiological procedures (BACTEC FX blood culture system; Becton, Dickinson and Co.). Contaminated blood cultures were defined according to previously suggested criteria [14]. Cases wherein bacteria were isolated regularly were referred to an infection medicine specialist for anti-bacterial treatment.

Patients were classified into non-infected and infected groups based on the absence and presence, respectively, of new-onset systemic bacterial infection.

### Psoas muscle area measurement

Abdominal CT images of patients scheduled for elective LDLT were assessed regularly within 1 month before surgery. The cross-sectional psoas muscle area (PMA) for lumbar vertebrae 3 and 4 was measured manually on two-dimensional abdominal CT (PACS Viewer; INFINITT Healthcare, Phillipsburg, NJ, USA) after removing intramuscular fat from the images using automated software (AQI; TeraRecon, Foster City, CA, USA). The average of the two PMA measurements was normalized to the patient’s height squared (psoas muscle index [PMI] = PMA × height^− 2^).

### Requirement of continuous renal replacement therapy

The kidney function of patients scheduled for elective LDLT was routinely checked by nephrologists, and patients with a severe decrease in kidney function before surgery (i.e., an increase in serum creatinine ≥4.0 mg/dL^− 1^ or to 3-fold of the baseline level, urine output ≤0.3 mL·kg^− 1^·h^− 1^ for 24 h, or anuria for 12 h) received continuous renal replacement therapy (CRRT) (Prismaflex system; Baxter, Deerfield, IL, USA) after central venous cannulation using a hemodialysis catheter (Power-Trialysis short-term dialysis catheter; Bard, New Providence, NJ, USA) [15, 16]. The dialysis catheters were inserted and handled according to the 2002 Centers for Disease Control and Prevention recommendations [17]. The catheter insertion site was disinfected using alcoholic povidone iodine [18]. Antimicrobial locks were not used in this study.

### Measurement of laboratory variables

As part of the preoperative evaluation, laboratory parameters, including neutrophil and lymphocyte counts, were measured in all patients scheduled for LDLT. All blood samples were collected without venous stasis into evacuated test tubes (BD Vacutainer, K2 EDTA; Becton, Dickinson and Co), and the parameters were measured using an automated hematology analyzer (XE-2100; Sysmex Corp., Kobe, Japan). If multiple tests were performed, the results obtained nearest to surgery were included in the analysis; combined parameters, such as the neutrophil to lymphocyte ratio (NLR), were calculated based on measurements obtained at the same time.

### Early allograft dysfunction

Early allograft dysfunction (EAD) was clinically defined as the presence of more than one of the following by POD 7: (1) total bilirubin ≥10 mg·dL^− 1^; (2) international normalized ratio (INR) ≥ 1.6; and (3) alanine or aspartate aminotransferase > 2000 IU·mL^− 1^. The definition of EAD used herein was validated in previous LT studies [19, 20].

### Perioperative recipient and donor-graft findings

Perioperative recipient data included age, sex, body mass index (BMI), PMI, etiologies for LDLT, comorbidities (diabetes mellitus and hypertension), model for end-stage liver disease (MELD) score, utility of CRRT, hepatocellular carcinoma (HCC) and HCC beyond the Milan criteria [21], hepatic decompensation (encephalopathy [West-Haven grade I or II] [22], varix and ascites), cardiac function (ejection fraction and diastolic dysfunction [23]), and laboratory variables (hemoglobin, white blood cell count, C-reactive protein to albumin [CRP/ALB] ratio, CRP, albumin, NLR, neutrophil count, lymphocyte count, platelet to lymphocyte ratio [PLR], platelet count, INR, and sodium, potassium, total bilirubin, creatinine, and glucose levels). Intraoperative recipient data included surgical duration, post-reperfusion syndrome [24], vital signs (mean blood pressure, heart rate, and central venous pressure [CVP]), mean lactate level, blood product transfusion (packed red blood cells [PRBCs], FFP and single donor platelets), hourly fluid infusion, and urine output. Donor-graft data included age, sex, BMI, graft-to-recipient weight ratio, ABO-incompatible graft, graft ischemic time, and graft fatty change. Early postoperative findings included the occurrence of EAD [19], acute kidney injury [15], biliary stricture or leakage, mechanical ventilation duration, and acute graft rejection and rejection activity index [25].

### Prognosis after LDLT

Postoperative outcomes included total duration of hospital and intensive care unit (ICU) stays and overall patient mortality.

### Statistical analysis

The normality of the distribution of the continuous data was evaluated using the Shapiro–Wilk test. The non-infected and infected groups were compared in terms of the perioperative recipient and donor-graft parameters using the Mann–Whitney *U* test and the *χ*^2^ or Fisher’s exact test, as appropriate. The linear-by-linear association method was used to analyze the data trends. The association between the perioperative clinical factors and early post-transplant bacteremia was analyzed by univariate and multivariate logistic regression. Significant factors, and those showing a trend toward significance (*p* < 0.1), in the univariate logistic analysis were entered into multivariate forward and backward logistic regression analyses. When multiple perioperative factors were inter-correlated, the most clinically relevant factors were retained in the models. The predictive accuracy of the models was evaluated according to the area under the receiver operating characteristic curve (AUC). The overall patient survival rate during the follow-up period was analyzed using the Kaplan–Meier method and compared between the two groups using the log-rank test. Values are expressed as medians with interquartile range (IQR) and numbers with proportions. All analyses were two-sided, and a *p* < 0.05 was considered significant. Statistical analyses were performed using SPSS for Windows (ver. 24.0; SPSS Inc., Chicago, IL, USA) and MedCalc for Windows software (ver. 11.0; MedCalc Software, Ostend, Belgium).

## Results

### Demographic characteristics of the patients undergoing LDLT

The study population included 419 male (70.3%) and 177 female (29.7%) patients. The median (IQR) age and BMI were 53 (48–59) years and 24.2 (22.1–26.6) kg·m^− 2^, respectively. The median MELD score was 15 (9–26) points. The etiologies for LDLT were: hepatitis B (53.9%); alcoholic hepatitis (23.0%); hepatitis C (6.5%); autoimmune hepatitis (4.5%); hepatitis A (4.2%); toxic hepatitis (2.7%); and cryptogenic hepatitis (5.2%).

The incidence of bacteremia 1 month after LDLT was 9.7% (57 patients). *Enterococcus faecium* (31.6%) was the bacteria most commonly cultured from blood samples, followed by *Acinetobacter baumannii* (10.5%), *Klebsiella pneumoniae* (10.5%), *Pseudomonas aeruginosa* (8.8%), vancomycin-resistant *Enterococcus* (8.8%), *Staphylococcus haemolyticus* or *epidermidis* (5.3%), methicillin-resistant *Staphylococcus aureus* (3.5%), and *Escherichia coli* (3.5%). Additionally, 10 patients (17.5%) suffered co-infections, including *Stenotrophomonas maltophilia*, *Enterobacter cloacae*, *Corynebacterium stratum*, and *Streptococcus sanguinis*. However, bacterial colonization of the dialysis catheter tip was not seen in any case. The median (IQR) interval between the end of surgery and the first occurrence of bacteremia was 12 (8–18) days among patients with a positive bacterial culture.

### Comparison of perioperative recipient and donor-graft parameters between the non-infected and infected groups

Patients with early post-transplant bacteremia had a lower preoperative PMI, higher MELD score, and greater requirement for CRRT that those without early post-transplant bacteremia (Table 1). The CRP/ALB ratio, CRP, NLR, lymphocyte count, and creatinine level were different between the two groups. Patients with early post-transplant bacteremia had a greater intraoperative requirement for PRBCs, higher hourly fluid infusion rate, and lower hourly urine output than those without early post-transplant bacteremia (Table 2). Patients with early post-transplant bacteremia were more likely to show EAD than those without early post-transplant bacteremia.

**Table 1:** Preoperative recipient findings in the non-infected and infected groups

Group	Non-infection	Infection	*p*
n	539	57	
Age (years)	53 (48–59)	54 (46–62)	0.845
Sex (male)	378 (70.1%)	41 (71.9%)	0.777
Body mass index (kg·m^-2^)	24.2 (22.1–26.6)	23.8 (21.3–25.7)	0.181
Psoas muscle index (mm^2^·m^-2^)	329.7 (261.0–401.4)	295.7 (226.8–370.9)	0.014
*Etiology*			0.543
Alcohol use	127 (23.6%)	10 (17.5%)	
Hepatitis A	21 (3.9%)	4 (7.0%)	
Hepatitis B	292 (54.2%)	29 (50.9%)	
Hepatitis C	33 (6.1%)	6 (10.5%)	
Autoimmune disorder	23 (4.3%)	4 (7.0%)	
Drugs and toxins	14 (2.6%)	2 (3.5%)	
Cryptogenic	29 (5.4%)	2 (3.5%)	
*Comorbidity*			
Diabetes mellitus	143 (26.5%)	12 (21.1%)	0.370
Hypertension	107 (19.9%)	11 (19.3%)	0.921
MELD score (points)	15 (9–26)	22 (13–35)	0.001
CRRT	67 (12.4%)	15 (26.3%)	0.004
Hepatocellular carcinoma	240 (44.5%)	19 (33.3%)	0.105
Beyond the Milan criteria	24 (22.0%)	1 (16.7%)	1.000
*Hepatic decompensation*			
Encephalopathy	50 (9.3%)	5 (8.8%)	0.900
Varix	132 (24.5%)	12 (21.1%)	0.564
Ascites	248 (46.0%)	33 (57.9%)	0.087
*Cardiac function*			
Ejection fraction (%)	64.6 (62.0–67.0)	64.6 (63.5–67.0)	0.174
Diastolic dysfunction	230 (42.7%)	25 (43.9%)	0.863
*Laboratory variables*			
Hemoglobin (g·dL^-1^)	9.8 (8.4–11.6)	9.2 (8.1–11.1)	0.243
WBC count (×10^9^·L^-1^)	4.4 (2.8–7.3)	4.4 (2.7–9.6)	0.689
C-reactive protein to albumin ratio	0.14 (0.04–0.54)	0.25 (0.07–0.88)	0.003
C-reactive protein (mg·dL^-1^)	0.4 (0.1–1.5)	0.8 (0.2–2.3)	0.005
Albumin (g·dL^-1^)	3.0 (2.6–3.4)	2.8 (2.6–3.4)	0.058
Neutrophil to lymphocyte ratio	2.80 (1.67–6.03)	3.99 (2.31–10.28)	0.002
Neutrophils (×10^9^·L^-1^)	2.5 (1.5–5.0)	2.8 (1.7–6.8)	0.131
Lymphocytes (×10^9^·L^-1^)	0.9 (0.6–1.4)	0.8 (0.4–1.2)	0.031
Platelet to lymphocyte ratio	76.09 (53.09–112.09)	81.27 (54.22–115.38)	0.583
Platelet count (×10^9^·L^-1^)	63.0 (45.0–102.0)	57.0 (37.5–86.0)	0.060
Sodium (mEq·L^-1^)	139 (135–142)	138 (134–141)	0.117
Potassium (mEq·L^-1^)	4.0 (3.7–4.3)	3.9 (3.6–4.5)	0.832
Total bilirubin (mg·dL^-1^)	2.5 (0.9–13.4)	2.8 (0.9–28.6)	0.204
International normalized ratio	1.5 (1.2–2.1)	1.5 (1.3–2.0)	0.777
Creatinine (mg·dL^-1^)	0.8 (0.7–1.2)	1.0 (0.7–2.0)	0.025
Glucose (mg·dL^-1^)	109 (92 –139)	113 (95–143)	0.555

*Abbreviations*: *CRRT* Continuous renal replacement therapy, *MELD* Model for end-stage liver disease, *WBC* White blood cell

NOTE: Values are medians (interquartile range) or numbers (%)

**Table 2:** Intraoperative recipient, donor-graft, and early postoperative parameters in the non-infected and infected groups

Group	non-Infection	Infection	*p*
n	539	57	
*Intraoperative recipient parameters*			
Surgical duration (min)	500 (450– 565)	515 (453–590)	0.364
Post-reperfusion syndrome	119 (22.1%)	19 (33.3%)	0.055
Mean vital sign values			
MBP (mmHg)	74 (68–80)	75 (70–79)	0.826
HR (beats·min^-1^)	90 (80–100)	93 (79–103)	0.363
CVP (mmHg)	9 (7–11)	10 (7–13)	0.146
Mean lactate (mmol·L^-1^)	3.7 (2.9–5.0)	3.6 (2.7–5.1)	0.493
*Blood product transfusion (unit)*			
Packed red blood cells	8 (4–13)	10 (5–21)	0.005
Fresh frozen plasma	7 (4–11)	8 (5–12)	0.102
Single donor platelet	1 (0–2)	1 (0–2)	0.208
Hourly fluid infusion (mL·kg^-1^·h^-1^)	9.6 (6.7–13.1)	10.5 (8.0–16.8)	0.012
Hourly urine output (mL·kg^-1^·h^-1^)	1.2 (0.6–2.0)	1.0 (0.2–1.7)	0.021
*Donor-graft parameters*			
Age (years)	35 (26–41)	35 (29–40)	0.346
Sex (male)	175 (32.5%)	19 (33.3%)	0.894
Body mass index (kg·m^-2^)	23.8 (21.9–25.3)	23.8 (22.1–25.1)	0.993
GRWR (%)	1.2 (1.0–1.5)	1.2 (1.0–1.6)	0.68
ABO-incompatible graft (%)	48 (8.9%)	7 (12.3%)	0.402
Graft ischemic time (min)	95 (70–128)	95 (67–137)	0.852
Graft fatty change (%)	4.7 (1.0–5.0)	4.7 (1.5–5.0)	0.138
*Early postoperative parameters*			
Early allograft dysfunction	64 (11.9%)	20 (35.1%)	<0.001
Acute kidney injury	157 (29.1%)	22 (38.6%)	0.142
Biliary stricture or leakage	81 (15.0%)	12 (21.1%)	0.233
Mechanical ventilation duration (min)	25 (0 – 765)	70 (0 – 711)	0.692
Acute graft rejection	109 (20.2%)	7 (12.3%)	0.15
Rejection activity index (score)^a^	4 (2 – 6)	3 (2 – 4)	0.071
Mild rejection (0 – 3 score)	50 (45.9%)	4 (57.1%)	0.843
Moderate rejection (4 – 6 score)	38 (34.9%)	2 (28.6%)	
Severe rejection (7 – 9 score)	21 (19.3%)	1 (14.3%)	

*Abbreviations*: *GRWR* Graft-recipient-weight-ratio, *MBP* Mean blood pressure, *HR* Heart rate, *CVP* Central venous pressure

^a^Rejection activity index in patients with acute graft rejection

NOTE: Values are medians (interquartile range) or numbers (%)

### Association between perioperative clinical findings and the occurrence of early post-transplant bacteremia

In univariate analysis, several preoperative recipient (PMI, MELD score, requirement for CRRT, ascites, CRP/ALB ratio, NLR, PLR, and sodium level), intraoperative recipient (post-reperfusion syndrome, mean CVP, requirement for PRBCs and FFP, hourly fluid infusion, and urine output), and short-term postoperative (EAD) parameters were associated with the risk of early post-transplant bacteremia (Table 3). In the multivariate analysis, PMI, requirement for CRRT, NLR, and EAD were independently associated with the risk of early post-transplant bacteremia (AUC: 0.707; 95% confidence interval: 0.667–0.745; *p* < 0.001).

**Table 3:** Association between perioperative recipient and donor-graft parameters and early post-transplant bacteremia in patients undergoing living donor liver transplantation

	Univariate analysis				Multivariate analysis			
	*β*	Odds ratio	95% CI	*p*	*β*	Odds ratio	95% CI	*p*
*Preoperative recipient parameters*								
Age (years)	−0.002	0.998	0.967–1.029	0.893				
Sex (female)	−0.087	0.916	0.500–1.680	0.777				
Body mass index (kg·m^-2^)	−0.057	0.945	0.876–1.019	0.142				
Psoas muscle index (mm^2^·m^-2^)	−0.004	0.996	0.993–0.999	0.017	−0.004	0.996	0.993–0.999	0.019
*Comorbidity*								
Diabetes mellitus	−0.303	0.738	0.380–1.436	0.371				
Hypertension	−0.035	0.965	0.484–1.927	0.921				
MELD score (point)	0.042	1.042	1.019–1.066	<0.001				
CRRT	0.923	2.516	1.323–4.784	0.005	0.710	2.034	1.004–4.125	0.049
Hepatocellular carcinoma	−0.473	0.623	0.350–1.108	0.107				
Beyond Milan criteria	−0.345	0.708	0.079–6.356	0.758				
*Hepatic decompensation*								
Encephalopathy	−0.061	0.940	0.359–2.463	0.900				
Varix	−0.196	0.822	0.422–1.601	0.565				
Ascites	0.478	1.613	0.929–2.803	0.090				
*Cardiac function*								
Ejection fraction (%)	0.036	1.036	0.973–1.104	0.267				
Diastolic dysfunction	0.048	1.050	0.605–1.820	0.863				
*Laboratory variables*								
Hemoglobin (g·dL^-1^)	−0.063	0.939	0.827–1.066	0.331				
WBC count (×10^9^·L^-1^)	0.009	1.009	0.965–1.054	0.701				
C-reactive protein to albumin ratio (%)	0.439	1.551	1.198–2.007	0.001				
Neutrophil to lymphocyte ratio (%)	0.052	1.053	1.020–1.087	0.002	0.032	1.032	1.000–1.066	0.048
Platelet to lymphocyte ratio	0.002	1.002	1.000–1.004	0.064				
Sodium (mEq·L^-1^)	−0.041	0.960	0.916–1.006	0.089				
Potassium (mEq·L^-1^)	0.009	1.009	0.645–1.579	0.968				
Glucose (mg·dL^-1^)	−0.001	0.999	0.994–1.004	0.796				
*Intraoperative recipient parameters*								
Surgical duration (min)	0.000	1.000	0.998–1.003	0.775				
Post-reperfusion syndrome	0.568	1.765	0.981–3.174	0.058				
*Mean vital sign values*								
MBP (mmHg)	−0.008	0.992	0.963–1.022	0.601				
HR (beats·min^-1^)	0.012	1.012	0.993–1.032	0.210				
CVP (mmHg)	0.092	1.097	1.011–1.190	0.026				
Mean lactate (mmol·L^-1^)	0.036	1.036	0.971–1.107	0.286				
*Blood product transfusion (unit)*								
Packed red blood cells	0.038	1.039	1.014–1.064	0.002				
Fresh frozen plasma	0.031	1.032	1.002–1.063	0.039				
Single donor platelets	0.015	1.015	0.916–1.126	0.774				
Hourly fluid infusion (mL·kg^-1^·h^-1^)	0.016	1.016	0.997–1.035	0.094				
Hourly urine output (mL·kg^-1^·h^-1^)	−0.250	0.779	0.589–1.029	0.079				
*Donor-graft parameters*								
Age (years)	0.011	1.011	0.988–1.036	0.354				
Sex (female)	0.039	1.040	0.583–1.856	0.894				
Body mass index (kg.m^-2^)	−0.003	0.997	0.911–1.091	0.945				
GRWR (%)	0.304	1.355	0.672–2.734	0.396				
ABO-incompatible graft	0.359	1.432	0.615 – 3.333	0.405				
Graft ischemic time (min)	0.001	1.001	0.998–1.004	0.653				
Fatty change (%)	0.025	1.025	0.993–1.059	0.125				
*Early postoperative parameters*								
Early allograft dysfunction	1.420	4.136	2.255–7.586	<0.001	1.199	3.315	1.721–6.388	<0.001
Acute kidney injury	0.421	1.523	0.866–2.680	0.144				
Biliary stricture or leakage	0.411	1.508	0.765–2.974	0.236				
Mechanical ventilation duration (min)	0.000	1.000	1.000 – 1.000	0.780				
Acute graft rejection	-0.594	0.552	0.244 – 1.252	0.155				

*Abbreviations*: *CRRT* Continuous renal replacement therapy, *MELD* Model for end-stage liver disease, *WBC* White blood cell, *GRWR* Graft-recipient-weight-ratio, *MBP* Mean blood pressure, *HR* Heart rate, *CVP* Central venous pressure, *CI* Confidence interval

### Prognosis according to the occurrence of early post-transplant bacteremia

**Fig. 1:**
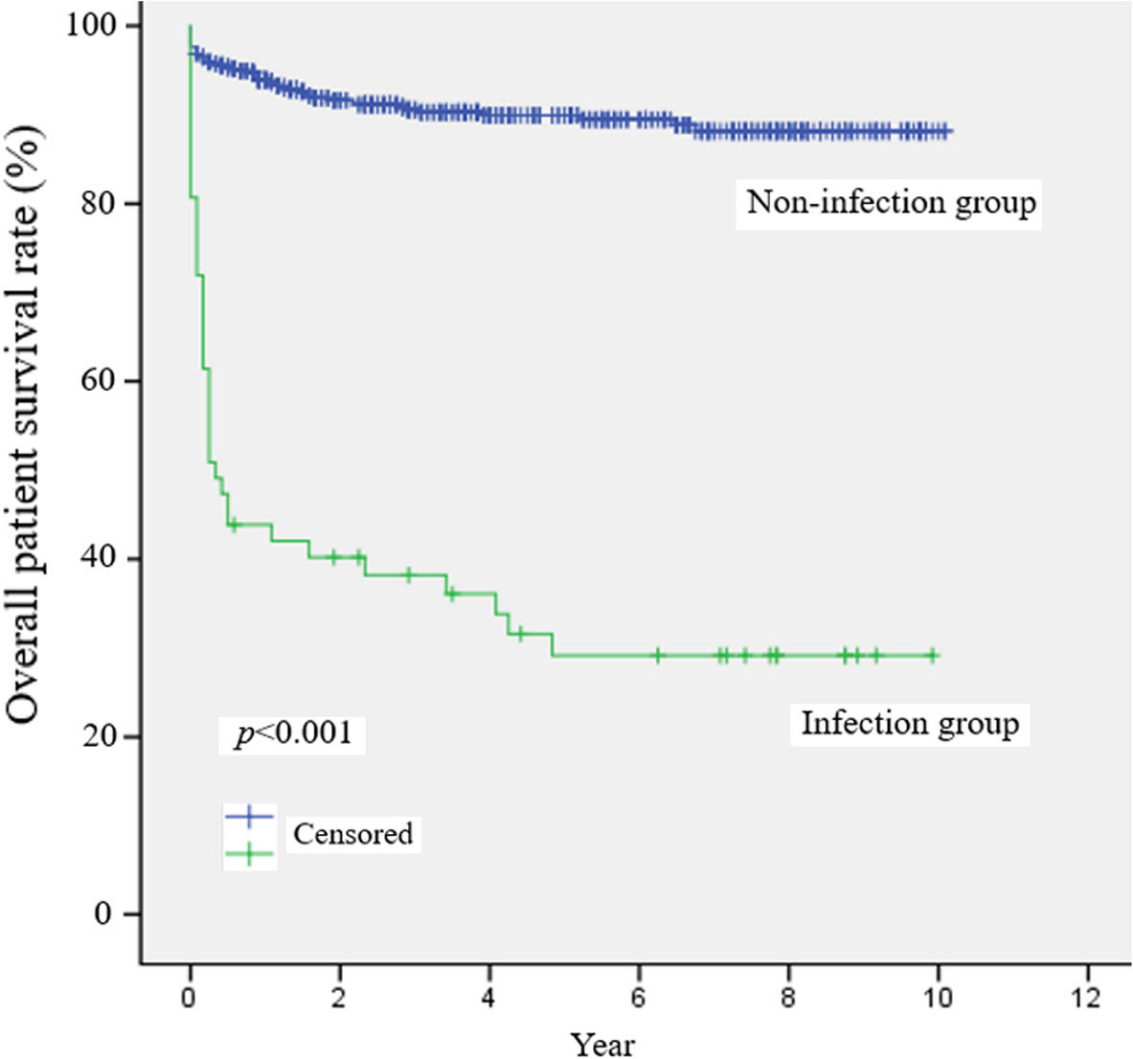
Comparison of overall survival between the non-infected and infected patient groups during the follow-up period after living donor liver transplantation. Overall patient survival was significantly different between the two groups (*p* < 0.001). The 1-, 3-, and 5-year survival rates were 93.9, 90.6, and 89.9% in the non-infected group, and 43.9, 38.2, and 29.1% in the infected group, respectively. The English in this document has been checked by at least two professional editors, both native speakers of English. For a certificate, please see: http://www.textcheck.com/certificate/r3kQzN

Compared to those without, patients with early post-transplant bacteremia had a longer median (IQR) hospital stay (26 [21–36] vs. 40 [31–56] days, respectively, *p* < 0.001) and ICU stay (7 [6, 7] vs. 13 [11–16] days, respectively, *p* < 0.001). The overall survival rate was higher in the non-infected group than in the infected group during the follow-up period (*p* < 0.001; Fig. 1). The 1-year survival rates were 93.9 and 43.9% in the non-infected and infected groups, respectively. The causes of post-transplant mortality included septic shock (*n* = 44; 48.9%), graft function insufficiency (*n* = 33; 36.7%), cancer (*n* = 11; 12.2%) and acute coronary syndrome (*n* = 2; 2.2%) (Additional file 1: Table S1). Among the 57 patients with early post-transplant bacteremia, infection was resolved in 23 patients (40.4%) after anti-bacterial treatment. However, infection persisted in 34 (59.6%) patients, leading to mortality in all of those cases (100.0%) (Additional file 2: Table S2). Additionally, among the factors included in the model (PMI, CRRT, NLR and EAD), EAD was significantly correlated with non-resolving infection in the 57 patients with early post-transplant bacteremia (Additional file 3: Table S3 and Additional file 4: Table S4).

## Discussion

The main finding of our study was that 57 patients (9.6%) suffered early postoperative bacteremia; the risk stratification model included preoperative recipient parameters (lower PMI, higher requirement for CRRT, and higher NLR) and postoperative graft parameters (development of EAD). Patients with an infection had longer hospital and ICU stays and a higher mortality rate than those without infection. Among patients with early post-transplant bacteremia, antibacterial treatment failed to resolve infection in 34 patients, resulting in increased risk of mortality. Among the factors included in the model, EAD was significantly correlated with non-resolving infection.

Skeletal muscle loss (i.e., sarcopenia) in critically ill patients, assessed using abdominal CT, is closely associated with an increased risk of mortality and/or morbidity, including infection [1, 26–30]. Skeletal muscle depletion is a major risk factor for perioperative infection in colorectal cancer surgery patients. Sarcopenia is related to a high prevalence of perioperative infection predominantly in older patients (aged ≥65 years) and delays patient recovery, as reflected in a higher likelihood of using rehabilitation care services and longer hospital stays [28]. Patients with sarcopenia undergoing restorative proctocolectomy for ulcerative colitis experienced more surgical site infections, which can result in pouch failure (i.e., persistent fistula and anal dysfunction), than those without sarcopenia [26]. Patients in the lowest tertile of total psoas area undergoing LT showed a 4-fold greater incidence of severe post-transplant infection than those in the highest tertile, and the infection had a negative impact on 1-year survival [31]. A lower psoas muscle area was associated with a higher risk of postoperative bacteria sepsis and lower overall patient survival in patients undergoing LDLT [32]. These findings mirror our result of a lower PMI (adjusted for sex) being independently associated with a high prevalence of early infection after surgery. Core muscle depletion is associated with aging, a lower level of physical activity, malnutrition, and consumptive diseases [33], and patients with a lower PMI may be more susceptible to postoperative infection.

Nosocomial bloodstream infection is a common complication of central venous catheter placement in patients admitted to the ICU [34]. ICU patients requiring CRRT have a higher risk for infection, and the hazard ratio for nosocomial bloodstream infection is 1.4-fold higher following CRRT [35, 36]. Independent of dialysis, kidney dysfunction is an important risk factor for sepsis, due to its association with the presence of uremic compounds (leptin, advanced glycation end products, and guanidine) that interfere with immune cells [37–40]. Cirrhotic LT patients frequently experience acute kidney injury and/or hepatorenal syndrome due to hepatitis virus, alcohol use, ascites, and hemorrhage [41]. Perioperative CRRT may help to control electrolyte levels and the acid-base balance without causing hemodynamic instability. Furthermore, a positive impact of CRRT on levels of ammonia and inflammatory mediators has been reported [42–45]. However, the association between pre- and intraoperative CRRT and early postoperative infection has not been investigated in detail in LDLT patients. In our study, patients with CRRT showed a 2-fold higher risk for a bloodstream bacterial infection than those without CRRT. Although the mechanism underlying the association of CRRT with infection is unknown, kidney disease etiology and CRRT type may be important. “Prophylactic” CRRT (e.g., monitoring for infection, providing preemptive antimicrobial treatment, ensuring sterility, etc.) may be helpful to reduce infection and severe sepsis risk in LT patients [46–48].

The NLR can easily be derived from the complete blood count [49] and is related to the prognosis of various diseases, including a number of cancers [50–53]. The NLR is also correlated with the severity of hepatic decompensation, as reflected in jaundice, ascites, and the MELD score in cirrhotic patients scheduled for LT, and is also independently associated with waiting list mortality after adjustment for the MELD score [54]. A higher NLR is associated with liver failure and mortality in patients on the waiting list for LT with a low MELD score (≤ 20 points) [55]. Stable cirrhotic patients without clinical signs or symptoms of endotoxemia experience a chronic subclinical inflammatory response, which increases the neutrophil count (reflected in demargination and retarded apoptosis of neutrophils, and stimulation of stem cells by growth factors) and decreases the lymphocyte count (reflected in margination, redistribution and apoptosis of lymphocytes) [54–57]. The NLR can be used to stratify patients with respect to the risk of hepato-circulatory dysfunction and the requirement for urgent LT. In chronic inflammatory diseases, such as coronary artery syndrome and peripheral artery disease, the NLR has been used as a biomarker of poor outcomes, such as arterial stiffness and a poor calcium score [58, 59]. Patients with a positive blood culture in an emergency care setting have a higher NLR than those with a negative blood culture. The NLR is more predictive of bacteremia than conventional infection markers (CRP, white blood cell count, and neutrophil count) [60]. Our results are similar to those of previous studies [54, 55, 60] with respect to a preoperative increase in the NLR being associated with a higher risk of early postoperative bacteremia and a higher mortality rate. The NLR was an independent predictor of early postoperative infection in our study after adjusting for other inflammatory markers.

Graft function recovery is important to satisfy the metabolic demands of cirrhotic patients undergoing LDLT [2]. EAD, which encompasses total bilirubin, the INR, aspartate transaminase (AST), and alanine aminotransferase (ALT) has been widely validated as an objective measure of post-transplant graft function [19, 20]. Our study is the first to show that EAD is associated with bloodstream bacterial infection during the first month after LDLT. Because the liver is located between the mesenteric and systemic circulation systems, and plays a key role in the defense against microbiological products and/or toxins emanating from the intestine [61], it is not surprising that EAD was associated with an increased risk of early postoperative bacteremia. Given the relationship between the development of EAD and early post-transplant infection, we suggest that the risk factors for EAD could serve as therapeutic targets to reduce the infection rate. Optimal donor and graft selection, in terms of age, BMI, fat type and extent, and graft size, may help to reduce the incidence of EAD and bacteremia in cirrhotic patients at high risk for bacteremia [19]. Additionally, patients with EAD seem to be vulnerable to post-transplant bacteremia, which is related to increased mortality. The optimal treatment regimen for bacteremic patients with EAD, including the type, infusion timing and dosage of drugs, has not yet been established, and anti-bacterial agents may have a negative impact on the liver (i.e., hepatotoxicity) [62]. Therefore, in patients with EAD, early identification of bacteremia and selection of appropriate and sensitive anti-bacterial drugs (ideally with lower hepatotoxicity) represents a good therapeutic strategy for preventing severe sepsis or septic shock.

Some limitations of our study should be discussed. Because patients undergoing LDLT are routinely administered prophylactic empirical antibiotics before and during surgery, false-negative bacterial culture results could have occurred after surgery, and the risk of early infection may have been underestimated. Also, we did not investigate the individual effect of each bacterium on prognosis. Additionally, because we only analyzed the bacteria in the systemic bloodstream, and not in the urine or sputum, the impact of infection on prognosis may have been underestimated. Although most patients were prescribed similar immunosuppression regimens according to our standard protocol, the clinical impact of regimen type was not considered. Also, we were unable to measure the nutritional status of the patients directly. Finally, we were unable to measure muscular strength (in the context of sarcopenia). Further study is required to investigate the association of sex-specific muscle mass depletion and weakness with the likelihood of early post-transplant infection.

## Conclusions

Newly occurring bacteremia during the early postoperative period had a negative impact on overall patient survival after LDLT. We propose a prognostic model to identify patients at high risk of bloodstream bacterial infection, and provide data supporting the notion that skeletal muscle depletion, CRRT requirement, systemic inflammatory response, and delayed liver graft function are associated with pathogenic vulnerability in cirrhotic patients undergoing LDLT.

## Supplementary information

**Additional file 1: Table S1.** Analysis of the cause of post-transplant mortality in 90 patients.

**Additional file 2: Table S2.** Analysis of post-transplant mortality rates according to infection resolution status in 57 patients with early post-transplant bacteremia.

**Additional file 3: Table S3.** Correlation of non-resolving infection with model factors in 57 patients with early post-transplant bacteremia.

**Additional file 4: Table S4.** Analysis of post-transplant infection status according to early allograft dysfunction (EAD) status in 57 patients with early post-transplant bacteremia.

## Data Availability

The datasets used and/or analyzed during this study are available from the corresponding author on reasonable request.

## Abbreviations

BMI: Body mass index
CRP: C-reactive protein
CRP/ALT ratio: C-reactive protein to albumin ratio
CRRT: Continuous renal replacement therapy
CT: Computed tomography
EAD: Early allograft dysfunction
ESLD: End-stage liver disease
FFP: Fresh frozen plasma
HCC: Hepatocellular carcinoma
INR: International normalized ratio
LDLT: Living donor liver transplantation
LT: Liver transplantation
MELD: Model fore end-stage liver disease
NLR: Neutrophil to lymphocyte ratio
PLR: Platelet to lymphocyte ratio
PMA: Psoas muscle area
PMI: Psoas muscle index
POD: Postoperative day
PRBC: Packed red blood cell
