# Supplementary material for: Risk stratification for early bacteremia after living donor liver transplantation: a retrospective observational cohort study
Source: BMC Surg. 2020 Mar 12;20:2. doi: 10.1186/s12893-019-0658-6 (PMC7066734; doi:10.1186/s12893-019-0658-6)
Supplement: Supplementary file 1 — Additional file 1: Table S1. Analysis of the cause of post-transplant mortality in 90 patients. [file 12893_2019_658_MOESM1_ESM.docx]

**Additional file 1**

| **Table S1.** Analysis of the cause of post-transplant mortality in 90 patients | |
| --- | --- |
| **Mortality causes** | **n=90** |
| Septic shock | 44 (48.9%) |
| Graft function insufficiency^*^ | 33 (36.7%) |
| Cancer occurrence | 11 (12.2%) |
| Acute coronary syndrome | 2 (2.2%) |
| ^*^Graft function insufficiency included graft failure and/or rejection.  **NOTE**: Values are expressed as number and proportions (%). | |
