# Supplementary material for: Risk stratification for early bacteremia after living donor liver transplantation: a retrospective observational cohort study
Source: BMC Surg. 2020 Mar 12;20:2. doi: 10.1186/s12893-019-0658-6 (PMC7066734; doi:10.1186/s12893-019-0658-6)
Supplement: Supplementary file 2 — Additional file 2: Table S2. Analysis of post-transplant mortality rates according to infection resolution status in 57 patients with early post-transplant bacteremia. [file 12893_2019_658_MOESM2_ESM.docx]

**Additional file 2**

| **Table S2.** Analysis of post-transplant mortality rates according to infection resolution status in 57 patients with early post-transplant bacteremia | | | |
| --- | --- | --- | --- |
| **Group** | **Resolving infection** | **Non resolving infection** | ***p*** |
| **n** | **23** | **34** |  |
| Survival | 18 (78.3%) | 0 (0.0%) | <0.001 |
| Non survival | 5 (21.7%) | 34 (100.0%)^†^ |  |
| ^†^*p*<0.001 using linear by linear association method  **NOTE:** Values are expressed as number and proportions (%). | | | |
