# Supplementary material for: Risk stratification for early bacteremia after living donor liver transplantation: a retrospective observational cohort study
Source: BMC Surg. 2020 Mar 12;20:2. doi: 10.1186/s12893-019-0658-6 (PMC7066734; doi:10.1186/s12893-019-0658-6)
Supplement: Supplementary file 3 — Additional file 3: Table S3. Correlation of non-resolving infection with model factors in 57 patients with early post-transplant bacteremia. [file 12893_2019_658_MOESM3_ESM.docx]

**Additional file 3**

| **Table S3.** Correlation of non-resolving infection with model factors in 57 patients with early post-transplant bacteremia | |
| --- | --- |
|  | **Non-resolving infection** |
| ***The factors in the model*** | Correlation coefficient |
| Psoas muscle index (mm^2^.m^-2^) | -0.179 |
| Continuous renal replacement therapy | 0.167 |
| Neutrophil to lymphocyte ratio (%) | 0.150 |
| Early allograft dysfunction | 0.305^‡^ |
| ^‡^*p*<0.05 using Spearman method | |
