# Supplementary material for: Risk stratification for early bacteremia after living donor liver transplantation: a retrospective observational cohort study
Source: BMC Surg. 2020 Mar 12;20:2. doi: 10.1186/s12893-019-0658-6 (PMC7066734; doi:10.1186/s12893-019-0658-6)
Supplement: Supplementary file 4 — Additional file 4: Table S4. Analysis of post-transplant infection status according to early allograft dysfunction (EAD) status in 57 patients with early post-transplant bacteremia. [file 12893_2019_658_MOESM4_ESM.docx]

**Additional file 4**

| **Table S4.** Analysis of post-transplant infection status according to early allograft dysfunction (EAD) status in 57 patients with early post-transplant bacteremia | | | |
| --- | --- | --- | --- |
| **Group** | **Non EAD** | **EAD** | ***p*** |
| **n** | **37** | **20** |  |
| Resolving infection | 19 (51.4%) | 4 (20.0%) | 0.021 |
| Non-resolving infection | 18 (48.6%) | 16 (80.0%)^††^ |  |
| ^††^*p*<0.05 using linear by linear association method  **NOTE:** Values are expressed as number and proportions (%). | | | |
